# Interactions of primaquine and chloroquine with PEGylated﻿ phosphatidylcholine liposomes

**DOI:** 10.1038/s41598-021-91866-0

**Published:** 2021-06-14

**Authors:** Andang Miatmoko, Ira Nurjannah, Nuril Fadilatul Nehru, Noorma Rosita, Esti Hendradi, Retno Sari, Juni Ekowati

**Affiliations:** Department of Pharmaceutical Sciences, Faculty of Pharmacy, Universitas Airlangga, Nanizar Zaman Joenoes Building, Campus C Unair, Mulyorejo, 60115 Indonesia

**Keywords:** Nanoscale materials, Other nanotechnology

## Abstract

This study aimed to analyze the interaction of primaquine (PQ), chloroquine (CQ), and liposomes to support the design of optimal liposomal delivery for hepatic stage malaria infectious disease. The liposomes were composed of hydrogenated soybean phosphatidylcholine, cholesterol, and distearoyl-*sn*-glycero-3-phosphoethanolamine-N-(methoxy[polyethyleneglycol]-2000), prepared by thin film method, then evaluated for physicochemical and spectrospic characteristics. The calcein release was further evaluated to determine the effect of drug co-loading on liposomal membrane integrity. The results showed that loading PQ and CQ into liposomes produced changes in the infrared spectra of the diester phosphate and carbonyl ester located in the polar part of the phospholipid, in addition to the alkyl group (CH_2_) in the nonpolar portion. Moreover, the thermogram revealed the loss of the endothermic peak of liposomes dually loaded with PQ and CQ at 186.6 °C, which is identical to that of the phospholipid. However, no crystallinity changes were detected through powder X-ray diffraction analysis. Moreover, PQ, with either single or dual loading, produced the higher calcein release profiles from the liposomes than that of CQ. The dual loading of PQ and CQ tends to interact with the polar head group of the phosphatidylcholine bilayer membrane resulted in an increase in water permeability of the liposomes.

## Introduction

Primaquine Primaquine (PQ) is the only effective anti-malarial used for the treatment of sporozoites in the hepatic phase of malaria infectious disease. However, it lacks efficacy against the asexual form of *Plasmodium* spp. in blood, indicating that it can not be used as a monotherapy, but should be administered in combination with blood schizonticides^1–3^. In addition, the use of PQ is limited by its tendency to cause serious side effects, including hemolysis in individuals deficient in glucose-6-phosphate dehydrogenase^1,2,4–6^. ‬Despite its currently limited therapeutic use because of widespread resistance^7–9^, the combined use of chloroquine (CQ), a blood schizonticide, reduces the toxicity of PQ while increasing its activity. A study by Fasinu et al.^10^ reported that CQ influences several metabolic pathways known to play a role in the activity and toxicity of PQ, encompassing the effect of hemolysis. In particular, CQ suppresses the number of metabolites generated through CYP2D6-mediated metabolism. Moreover, CQ changes the disposition and pharmacokinetic profiles of PQ, resulting in higher drug levels and tissue exposure^11^. In addition, the combination is also used clinically in the treatment of *Plasmodium vivax* malaria, and CQ also enhances the sensitivity of *Plasmodium falciparum* to PQ^12^‬. The use of liposomes as drug delivery carriers increases the activity of anti-malaria drugs^13^. However, PQ is known to influence the structure of the liposomal bilayer membrane. Basso et al.^14^ reported the existence of an electrostatic interaction between the negative charges of a phosphate group on the polar phospholipid portion of dimyristoylphosphatidylcholine (DMPC) and a positive nitrogen charge in the PQ structure. Furthermore, a hydrophobic interaction also occurs between the quinoline ring of PQ and the hydrocarbon chain of DMPC^14,15^. Therefore, the existence of these two interactions leads to the insertion of PQ into the DMPC structure, thereby disrupting the arrangement and dynamics of the acyl chain rotation and resulting in enhanced fluidity of the bilayer membrane. Conversely, it was reported that CQ induces the opposite effect via its interaction with the polar part of dipalmitoylphosphatidylcholine (DPPC), causing the absorption of CQ molecules on the surface of the liposomes^15–17^. This inhibits the movement of the acyl chain, consequently enhancing the rigidity of the bilayer membrane^16^.

Changes in liposome membrane rigidity affect drug release^18,19^, as denoted by the more rapid drug release from egg yolk phosphatidylcholine, which possesses a more fluid structure, than from the relatively rigid DPPC^20^. Moreover, lipids constituted in a non-rigid liposomal membrane often cause leakage of entrapped drugs, a condition known to affect the therapeutic index^18,19,21^. In this case, membrane rigidity is influenced by the characteristics of the lipid composition employed, as well as the addition of cholesterol to the exterior of the liposomal membrane^21,22^. Furthermore, drug release is potentially influenced by the presence of precipitation or the aggregation of drugs in the liposomes^23^. A previous study reported the tendency for colloidal aggregate formation between drugs and polymers in liposomes which can slow drug release^24^.

Combining two or more drugs within the same nanocarrier using a dual loading technique can control the drug release rate, thereby affecting the biodistribution and metabolism of each drug^25^. A previous study under taken by the authors of this article revealed that the dual loading of PQ and CQ significantly influenced the efficiency of drug trapping and release^26^. In single-loaded liposomes, the encapsulation efficiencies were 72% ± 4% for PQ and 56% ± 15% for CQ, whereas in co-loaded liposomes, they were 6% ± 1% and 31% ± 2%, respectively. In addition, liposomes co-loaded with PQ and CQ exhibited relatively slower drug release than those loaded with either drug alone. It has been reported that the encapsulation of two drugs in the same nanocarrier can modify the release profile of each when they both interact with the bilayer membrane^27^. Optimal delivery to hepatocytes should constitute the main objective when treating a malarial sporozoite invasion. Both positive therapeutic effects and reduced hemolysis in cases of patients suffering from glucose-6-phosphate dehydrogenase deficiency should be produced. Therefore, the use of liposomes as drug carriers is indispensable, rendering an effective strategy for further liposome formulation essential in order to achieve high and stable drug encapsulation.

In the current study, the effect of dual-loaded PQ and CQ on the integrity of the liposomal bilayer membrane was analyzed in relation to changes in membrane rigidity. This evaluation involved determining the physicochemical characteristics and release profile of the fluorescent compound calcein as indicators of membrane leakage^28–30^. Calcein was used because of the ease with which it is entrapped in the aqueous intraliposomal phase because of its low Log *P* value. In addition, calcein is hydrophilic and exhibits no interaction with the liposomal membrane^31^. Analyzing the integrity of the liposomal bilayer membrane is extremely important for observing the level of carrier leakage which is positively correlated with stability during distribution through systemic circulation before reaching the hepatocytes. It is anticipated that the data obtained will prove useful for evaluating changes in the membrane structure of liposomes containing PQ and CQ.

## Results and discussion

This study aimed to provide information related to the effect of PQ and CQ co-loading on the integrity of the bilayer membrane of liposomes. This analysis should be beneficial for designing optimal PQ and CQ delivery systems for malaria therapy, especially with regard to the hepatic phase. The liposomes were analyzed to determine their physicochemical characteristics and assess their calcein release profiles to confirm the integrity of the liposomal membrane. The physicochemical characteristics were specifically, evaluated using FTIR spectroscopy, P-XRD, and DTA. These evaluations were performed to analyze the interaction between the drugs and the lipid membrane of the liposomes^32,33^.

In a previous study, the drug-to-lipid ratios of PQ and CQ were optimized during the drug loading process^26^, in consideration of the dose ratios of both drugs in clinical practice^34–36^. In this study, a saturated phospholipid, i.e. HSPC, was used as the lipid component and the liposomes were prepared under equal conditions. Moreover, citrate buffer pH 5.0 was employed as the hydrating solution since low pH may result in hydrolysis of phospholipid. In the previous report referred to above, a change in pH of the citrate buffer from 4.0 to 5.0 reduced drug loading with the result that only 35% of PQ and 69% of CQ was encapsulated in the liposomes^37,38^. Moreover, the report showed that incubating the mixtures at a higher temperature than T_m_ of phospholipid, i.e. 60 °C, reduced the encapsulation efficiencies of PQ and CQ due to the increase in membrane water permeability causing a decrease in pH gradient during heating^37,38^. All liposomes were similar with regard to particle size, PDI, and ζ-potential, reflecting the fact that loading PQ and CQ had no significant effects on their physical characteristics. However, when PQ was dually loaded with CQ into liposomes, the encapsulation efficiency decreased significantly, as shown in the authors’ previous study^26^. However, there were differences in their spectroscopic and crystallinity profiles, as demonstrated by the findings of this study.

### Physical characteristics of the liposomes

The particle size, PDI, ζ-potential, drug encapsulation efficiency, and loading capacity of Lipo-PQ, Lipo-CQ, and Lipo-PQCQ are presented in Table 1. The results revealed no significant differences in the obtained values, as particle size, PDI, and ζ-potential ranging from 114.0 to 130.8 nm, 0.24–0.31, and − 16.38 to − 12.33 mV, respectively. Following dual loading of these two drugs, the encapsulation efficiencies decreased significantly from approximately 80 to 7% and 54 to 31% respectively for PQ and CQ. The loading capacities were approximately 16% and 6% for Lipo-PQ and Lipo-CQ, but there were significant reductions in the amounts of PQ and CQ loaded into Lipo-PQCQ which stood at only 1% and 4% in each case. While PQ is soluble in water^39^, its solubility is still almost 10–30 times lower than that of CQ which is categorized as freely water soluble^40^. The solubility of both drugs has been known to be affected by pH^39,40^. The contrasting solubility probably influences the intraliposomal physical condition of drugs after active loading using a pH gradient which may affect the drug loading. However, in this study, the drugs i.e. PQ and CQ were added as drug solutions in PBS pH 7.4 (the outer liposomal phase) with citrate buffer pH 5 as the intraliposomal phase, in order to ensure that solubility exerts a minimal influence on the research results. Moreover, the transmission electron microscopy (TEM) images reveal that no drug aggregates were observed inside the liposomes, as presented in Supplementary Fig. S1. These results show that both PQ and CQ are still soluble in the intraliposomal media, thus providing no or minimal effects of drug solubility on membrane integrity. These results were similar to those of the previous study^26^ proving that dual loading of PQ and CQ affects drug encapsulation without changing particle size or ζ-potentials. Consequently, physicochemical analysis is required. Having identified the typical interactions, appropriate further courses of action would be decided on for optimal dual delivery of PQ and CQ in cases of malaria.

**Table 1:** Characteristics of liposomes loaded with primaquine/PQ (Lipo-PQ), chloroquine/CQ (Lipo-CQ), and both drugs (Lipo-PQCQ) following incubation at 60 °C for 20 min. Each value represents the mean ± SD (n = 3).

Formula	Particle size (nm)	Polydispersity index (PDI)	ζ-Potential (mV)	Encapsulation efficiency (%)	Loading capacity (%)
Lipo-PQ	114.0 ± 4.2	0.24 ± 0.04	− 12.33 ± 2.98	80.65 ± 11.26	16.50 ± 3.70
Lipo-CQ	123.4 ± 5.9	0.31 ± 0.01	− 16.38 ± 3.91	54.56 ± 10.59	6.05 ± 0.97
Lipo-PQCQ	130.8 ± 8.3	0.31 ± 0.01	− 12.33 ± 2.98	7.17 ± 2.25 (PQ)	1.02 ± 0.37 (PQ)
				31.78 ± 3.85 (CQ)	4.52 ± 0.63 (CQ)

### Analysis of the physicochemical characteristics of the liposomes

#### FTIR profiles of the liposomes

**Figure 1:**
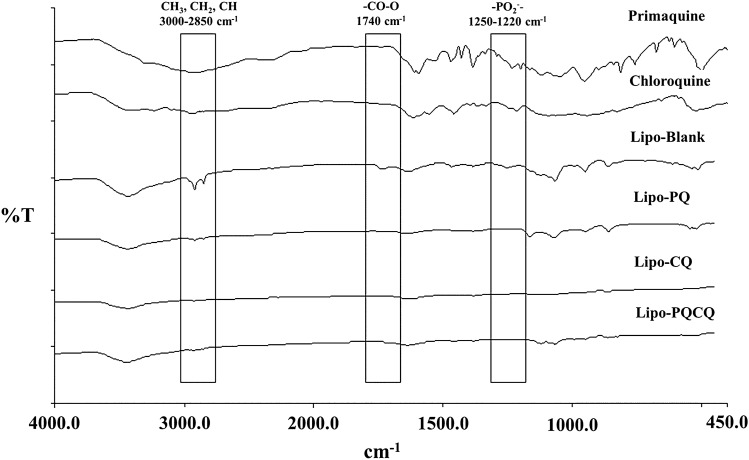
Fourier-transform infrared spectra of primaquine, chloroquine, blank liposomes (Lipo-Blank), primaquine-loaded liposomes (Lipo-PQ), chloroquine-loaded liposomes (Lipo-CQ), and liposomes loaded with both primaquine and chloroquine (Lipo-PQCQ) analyzed using the KBr pellet method.

In this study, FTIR analysis was used to determine the interactions of PQ and CQ with the liposomal membrane by observing the absorption band in the wavenumber ranges of particular functional groups. This included the diester phosphate (R–PO_2_–R′) and carbonyl ester (R–CO–O–R′) located in the polar part of the HSPC phospholipid, as well as the alkyl group (CH_2_) in the nonpolar portion^41,42^. Lipo-PQ, Lipo-CQ, and Lipo-PQCQ were analyzed for their spectra identification using FTIR, and their profiles were compared with those of Lipo-Blank, free PQ, and free CQ. As shown in Fig. 1, in accordance with the wavenumbers of each functional group listed in Table 2, variations in the absorption intensity of functional groups were observed among the liposomes. The FTIR spectrum of Lipo-PQ exhibited absorption bands with reduced intensities in the wavenumber ranges of carbonyl ester and diester phosphate groups compared with the findings for Lipo-Blank. It has been reported that PQ interacts electrostatically with lipid polar head group causing local acyl chain disorder and less densely packed bilayer membrane gel. Moreover, the quinoline ring of PQ inserts between the acyl chain of the hydrophobic tail of phospholipids causes membrane fluidity^14,15^. Therefore, the primaquine has probably been completely concealed inside the liposomes, producing the similar FTIR spectra of the Lipo-Blank and Lipo-PQ.

**Table 2:** The peak absorbance value of the infrared spectra of free primaquine (PQ), free chloroquine (CQ), blank liposomes (Lipo-Blank), primaquine-loaded liposomes (Lipo-PQ), chloroquine-loaded liposomes (Lipo-CQ), and liposome co-loaded with primaquine and chloroquine (Lipo-PQCQ).

Functional groups	Wavenumber (cm^−1^)						
	Ref	Prima-quine	Chloro-quine	Lipo-Blank	Lipo-PQ	Lipo-CQ	Lipo-PQCQ
O–H/N–H stretching	3550–3200	3297	3411; 3236	3442	3441	3441	3437
CH_3_, CH_2_, CH stretching	3000–2850	2968; 2945; 2883	2969; 2935; 2850	2956; 2919; 2851	2920; 2851	2924	2923
R–CO–OR’ (carbonyl ester)	1740	–	–	1738	1739	–	–
C=C stretching	1630–1680	–	–	1636	1636	1632	1638
C–C ring stretching (quinolone)	1612	1612	1614	–	–	–	–
C–N stretching	1558	1533	1552	–	–	–	–
CH_2_, CH_3_ bending	1470–1350	1469; 1430; 1385	1458; 1393; 1368	1467; 1384	1384	1458; 1384	1457; 1384
R–PO_2_^−^ –R’ (diester phosphate)	1250–1220	1234	1245	1254	1253	–	–
C–O stretching	1250–970	1165	1132	1120	1165	1121	1121
P–O Asymmetric stretching	1058	1050	1065	1067	1070	1074	1066
N^+^–CH_3_ (choline)	970	–	–	–	–	–	–
=C–H, =CH_2_	995–880	953;899	942; 907; 881	951; 863	950; 861	953; 863	952; 865

Meanwhile, Lipo-CQ and Lipo-PQCQ featured no phosphate and carbonyl group bands at 1740 cm^−1^ and 1230 cm^−1^, which reflect the interfacial and head region of the bilayer membrane^43^. Hence, it was assumed that interaction probably occurred between the drugs and liposomes rendering them undetectable.

Furthermore, the CH_3_, CH_2_, and CH bonds of Lipo-CQ and Lipo-PQCQ exhibited weaker absorption bands than those of Lipo-Blank and Lipo-PQ. The absorption band of the alkyl group possibly serves as an indicator of the lipid sequence which reflects the order of arrangement. The shift to a higher wavenumber, reduction in intensity, and widening of the absorption band were indicative of an increase in the gauche conformation of the aliphatic lipid chain^42^. The FTIR spectrum also revealed a decline in intensity of Lipo-PQ compared with those of Lipo-CQ and Lipo-PQCQ, indicating an increase in the gauche conformation of its hydrocarbon chain. Thus, the arrangement and density of the hydrocarbon chain had changed, possibly reflecting increased membrane fluidity^44^.

#### P-XRD profiles of the liposomes

**Figure 2:**
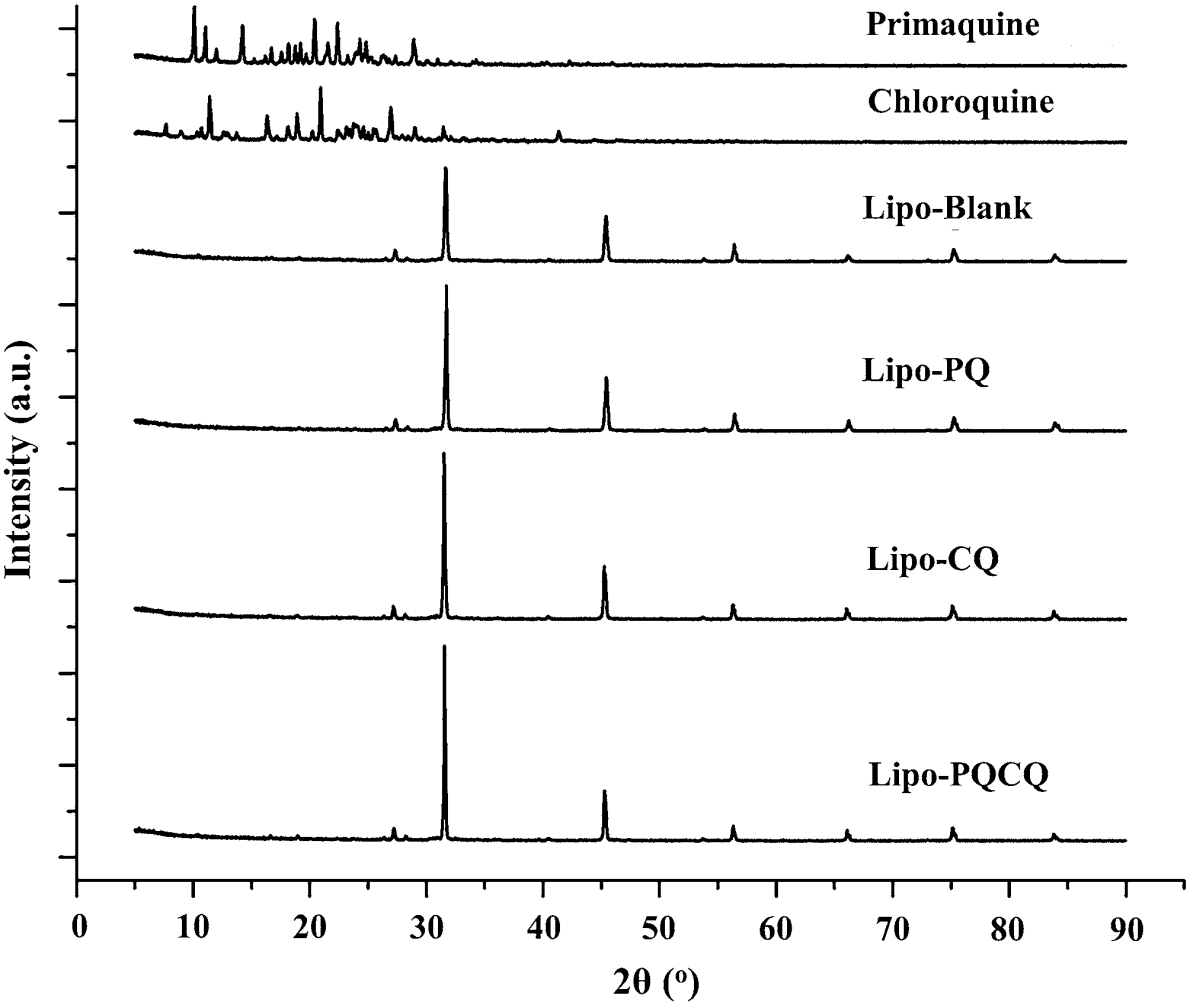
Powder X-ray diffraction analysis of primaquine, chloroquine, blank liposomes (Lipo-Blank), primaquine-loaded liposomes (Lipo-PQ), chloroquine-loaded liposomes (Lipo-CQ), and liposomes loaded with both primaquine and chloroquine (Lipo-PQCQ).

The diffractograms of the liposomes were obtained using P-XRD. As shown in Fig. 2, the P-XRD pattern of the free PQ and free CQ exhibited several sharp peaks indicative of crystallinity. However, these crystal patterns were absent from the diffractograms of Lipo-PQ, Lipo-CQ, and Lipo-PQCQ, as well as Lipo-Blank. The X-ray diffractogram patterns of liposomes displayed sharp peaks showing a high degree of crystallinity and all these samples had peaks identical to one another, indicating a similar degree of crystalinity.

#### DTA profiles of the liposomes

**Figure 3:**
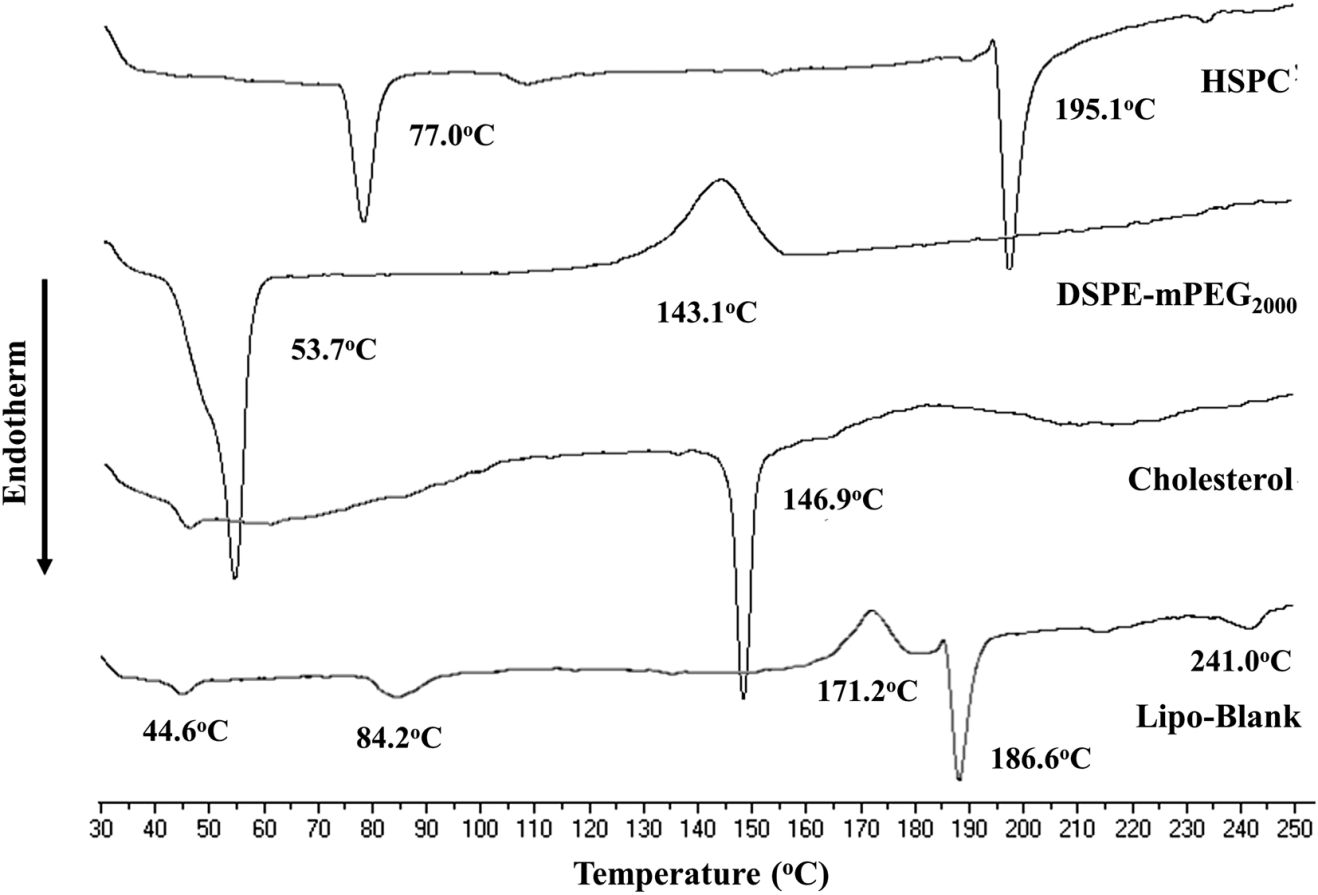
Differential thermal analysis of hydrogenated soybean phosphatidylcholine (HSPC), distearoyl-sn-glycero-3-phosphoethanolamine-N-(methoxy[polyethylene glycol]-2000) (DSPE-mPEG2000), cholesterol, and blank liposomes (Lipo-Blank).

**Figure 4:**
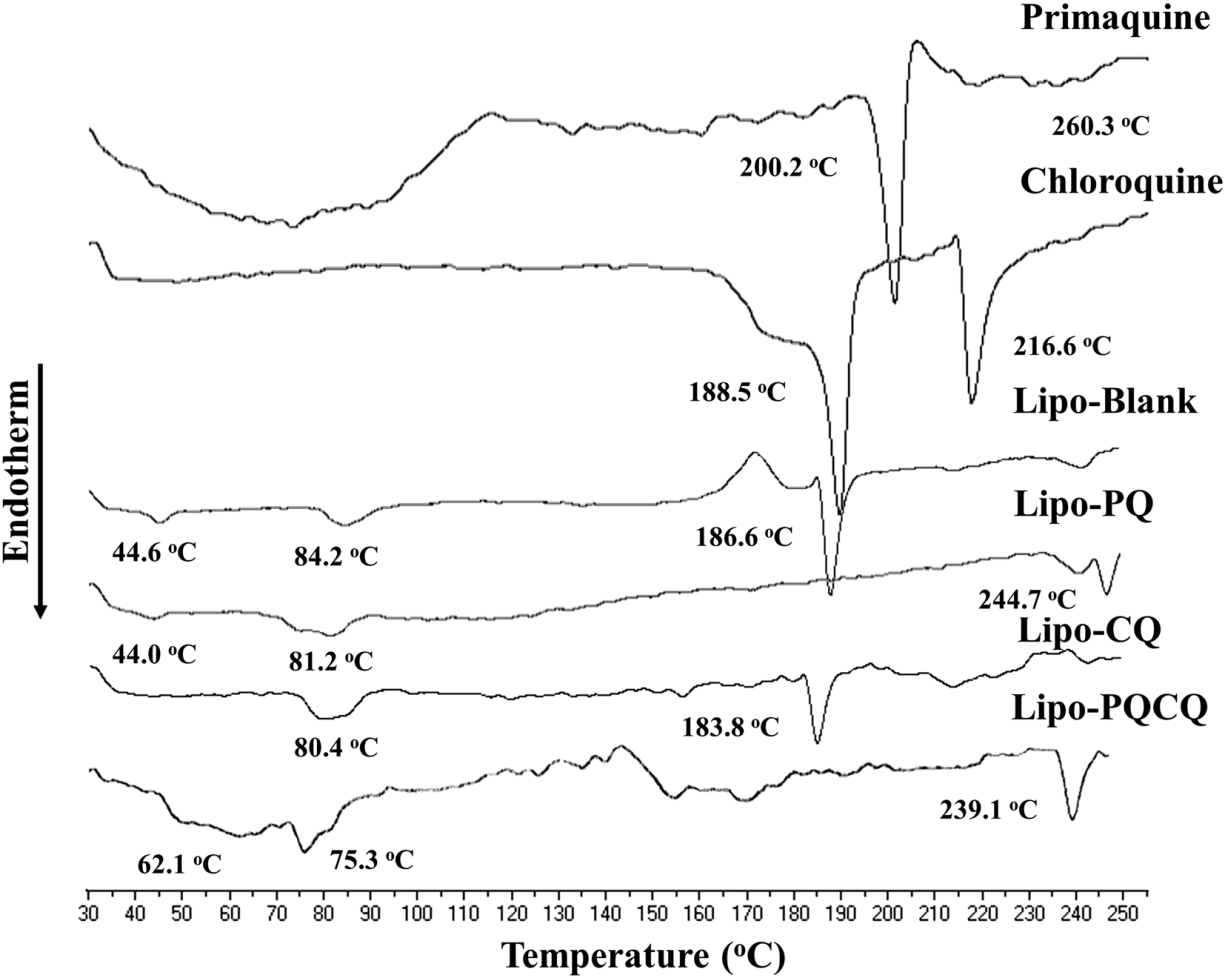
Differential thermal analysis of blank liposomes (Lipo-Blank), primaquine-loaded liposomes (Lipo-PQ), chloroquine-loaded liposomes (Lipo-CQ), and liposomes loaded with both primaquine and chloroquine (Lipo-PQCQ).

In addition, the effects of PQ and CQ on the changes in the physical properties of liposomes were also supported by the DTA thermograms. The DTA results of liposomal membranes are contained in Figs. 3 and 4. As shown in Fig. 3, two endothermic peaks were observed for HPSC at 77.0 °C and 195.1 °C, one endothermic peak and one exothermic peak were found for DSPE-mPEG_2000_ at 53.7 °C and 143.1 °C, respectively, and a single endothermic peak was identified for cholesterol at 146.9 °C. Moreover, endothermic peaks were identified at 44.6 °C, 84.2 °C, 186.6 °C, and 241.0 °C for Lipo-Blank. Meanwhile, following liposome formation, the peaks in the Lipo-Blank thermogram were identical to those of each of the lipid components, although some melting point shifts were identified.

Figure 4 presents the DTA thermograms of drug-loaded liposomes, free PQ, and free CQ. PQ had a sharp endothermic peak at 200.2 °C and a broad peak at 71.0 °C. Conversely, two sharp endothermic peaks were found at 188.5 °C and 216.6 °C for CQ. The drug-encapsulated liposomes displayed significant changes compared with those of the free drugs. Lipo-PQ had no identical endothermic peak to that of PQ. Moreover, compared with the findings for Lipo-Blank, the endothermic peak at 186.6 °C was not present, while a new endothermic peak appeared at 244.7 °C. There were also peak shifts at 44.0 °C and 81.2 °C. Moreover, in the thermogram of Lipo-CQ, the endothermic peak at 44.0 °C had disappeared, whereas an identical peak observed for Lipo-Blank had shifted to 183.8 °C. Lipo-CQ also had no identical peak to that of CQ, but a weak broad endothermic peak appeared at approximately 210 °C.

Meanwhile, Lipo-PQCQ experienced broad endothermic peaks at 62.1 °C and 75.3 °C and a sharp peak at 239.1 °C. However, the peak at 186.6 °C was absent. The thermogram peaks of Lipo-PQCQ were identical to those of PQ and Lipo-PQ.

There were shifts and losses of endothermic peaks in both the Lipo-PQ and Lipo-CQ thermograms compared with the findings in the Lipo-Blank thermogram. Furthermore, the endothermic peak displayed a major transition of the crystalline gel-liquid phase within the liposomes which is gelatinous at higher temperatures^45^. In addition, the Lipo-PQCQ thermogram demonstrated the loss of the endothermic peak at 186.6 °C, identical to that for HSPC observed in the Lipo-Blank thermogram. The loss of the endothermic peak identical to that of HSPC was also observed in the Lipo-PQ thermogram, but not in the Lipo-CQ thermogram**.** The loss or decline of this peak is indicative of an increase in the distance between membranes which can reduce the strength of the phospholipid arrangement in the gel phase^14,15^. This change is usually accompanied by a reduction in membrane rigidity together with decreased van der Waals bonds between acyl chains and phospholipids^45,46^. Therefore, Lipo-PQCQ may experience diminished strength of its phospholipid arrangement as denoted by a liquid phase, resulting in increased fluidity of the bilayer membrane of the liposomes.

#### NMR spectra of liposomes

Data obtained via NMR analysis are proton signals which include chemical shifts and multiplicity, as well as signal integration. From the results of ^1^H NMR, both CQ and PQ experienced interactions with liposomes.

**Figure 5:**
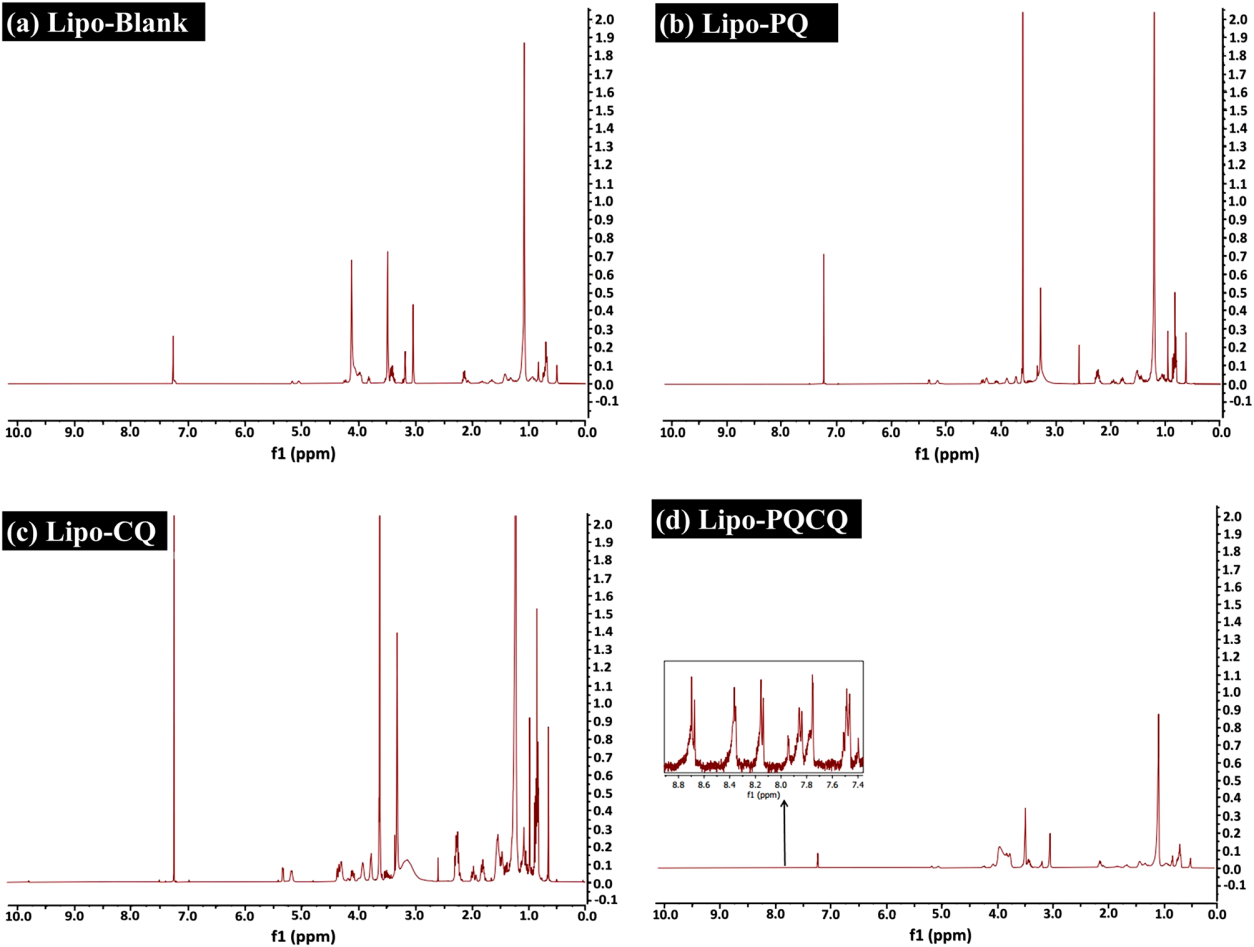
^1^H nuclear magnetic resonance spectra of blank liposomes (Lipo-Blank), primaquine-loaded liposomes (Lipo-PQ), chloroquine-loaded liposomes (Lipo-CQ), and liposomes loaded with both primaquine and chloroquine (Lipo-PQCQ) in CDCL_3_ at 400 MHz.

The ^1^H NMR spectrum of Lipo-Blank featured chemical shifts at 3.02 (s, 9H) and 3.82 ppm (m, 2H), which reflected the proton signals of C_13_ and C_14_, respectively, which bound to N atoms in the polar head of lipids in the bilayer (Fig. 5A). Moreover, the signals of C_20,20′_ bound to the CH_3_ group underwent a chemical shift at 0.70 ppm (m, 3H). The proton signals of alkyl (C–C) groups bound to an acyl group was observed for C_1-10_ and C_1′-10′_ with a chemical shift at 1.09 ppm (m, 40H). In addition, the proton signals of C_19_ and C_19′_ experienced chemical shifts at 2.14 (m, 2H) and 1.41 ppm (m, 2H), respectively. In addition, the proton signals of C_12_ and C_12′_ appeared at 2.01 (m, 2H) and 1.67 ppm (m, 2H), respectively. These signals reflect the hydrocarbon chain as the hydrophobic portion of the lipid bilayer. C–O groups which were observed at C_15_, C_16_, C_17_, and C_18_, appeared as chemical shifts at 5.10 (m, 1H), 4.10 (m, 2H), 3.48 (m, 2H), and 3.40 ppm (m, 2H), respectively.

However, the NMR spectra of Lipo-PQ and Lipo-CQ were similar to those of Lipo-Blank, as shown in Fig. 5B and C. However, dual loading of PQ and CQ into liposomes resulted in the appearance of the proton signals of the aromatic quinoline ring of PQ, whereas that of CQ had weak signal intensity, as shown in Fig. 5D. Some proton signals were noted for the aromatic ring of PQ. The proton signals of C_1_ bound to N and C_2_ atoms appeared as chemical shifts at 8.53 (m, 1H) and 7.49 ppm (m, 1H), respectively. Moreover, the chemical shift at 8.16 ppm (d, J = 7.1 Hz, 1H) indicated that C_3_ was coupled to the proton of C_2_. The proton signals of C_4_ and C_6_ in the aromatic ring appeared as chemical shifts at 7.49 (m, 1H) and 6.65 ppm (d, J = 7.3 Hz, 1H), respectively, coupled with N7, whereas the C_5_ methoxy group was represented by the chemical shift at 3.07 (s, 3H). The N7 proton signal appeared as a chemical shift at 6.25 ppm (m, 1H). The NMR spectra provided evidence that the signal intensity of the aromatic quinoline ring was stronger for PQ than for CQ. This indicates that PQ had an important role in the fluidity of the membrane.

#### Profile of calcein release from liposomes

**Figure 6:**
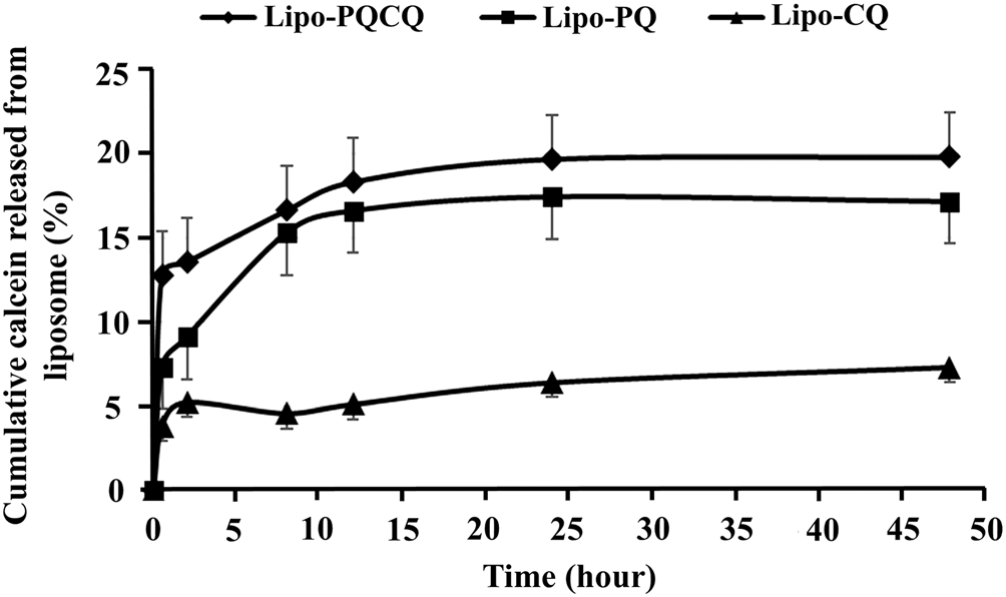
Profiles of calcein release from primaquine-loaded liposomes (Lipo-PQ), chloroquine-loaded liposomes (Lipo-CQ), and liposomes loaded with both primaquine and chloroquine (Lipo-PQCQ) in phosphate-buffered saline (pH 7.4) at 37 °C.

The effect of dual drug loading on membrane integrity was supported by the profiles of calcein release from the liposomes which was higher for Lipo-PQCQ and Lipo-PQ than for Lipo-CQ, as presented in Fig. 6. The results illustrated that the Lipo-CQ had the lowest percent calcein release of 7% after 48 h. Conversely, the percent calcein release for Lipo-PQ and Lipo-PQCQ was relatively similar, ranging from 17 to 20%. It is known that drug release from liposomes increases with increasing fluidity of the membrane^31^. Therefore, it has been established that the dual loading of PQ and CQ affected the fluidity of liposomes.

The physicochemical characteristics of the liposomal membrane and calcein release profile revealed that Lipo-PQ had relatively higher fluidity than Lipo-CQ leading to increased calcein release. Furthermore, the decreased integrity observed in Lipo-PQCQ was attributable to the increased membrane fluidity resulting from the interactions of PQ and CQ with the phospholipid bilayer. However, in a previous study, dual loading of PQ and CQ resulted in slower drug release compared with that in single drug-loaded liposomes^26^. Calcein is a polyanionic molecule with negative surface potential charges which mainly diffuses through the phospholipid bilayer^31^. The burst release of calcein during the first hour of this study could be due to the higher amount of unionized calcein molecules in the low pH of the intraliposomal phase containing citrate buffer pH 5.0^47^. This would, in turn, cause the release of a larger amount of calcein in the outer phase. The previous study of calcein release from liposomes also indicated that approximately 20–25% of calcein release from liposomes occurs in buffer pH 7.4. This is lower than that the 40–50% released from the liposome in pH 4.0 over a period of 48 hours^48^. However, due to the high intrinsic permeability of protons through the lipid bilayer, H^3+^ ions will permeate from the acidic intraliposomal phase to the exterior until a state of equilibrium is reached. This resulted in no further calcein release to that observed in the early phase^49,50^. It has been reported that calcein release is limited by lipid packing order^31^ and drug interaction within the bilayer membrane^51^. The stronger the interaction between the drug and lipid, the less calcein will be desorbed leading to burst effects. In this study, the interaction between PQ, CQ, and liposomes was observed, resulting in burst release of calcein from liposomes.

Moreover, as confirmed by the NMR and FTIR spectra, interaction could occur between PQ, CQ, and liposomes resulting in similar calcein release profiles of Lipo-PQ and Lipo-PQCQ, although the use of CQ could rigidify the bilayer membrane^16,52^. This may be attributable to the differences in chemical characteristics between calcein and PQ/CQ. Further research evaluating the molecular interaction and changes in liposomes structure is required to confirm the results of this study. These findings will provide some insights into the design of liposomes for delivering the combination of PQ and CQ specifically for hepatic stage malaria.

## Conclusions

As delivery of PQ in thes early stages of sporozoite invasion of the liver largely determines the success of preventing blood stage malaria infection, a strategy combining PQ load with CQ, a blood schizontocide, in liposomes offers strong therapeutic efficacy as well as reduced drug toxicities. However, this study reveals that dual drug loading of PQ and CQ into PEGylated liposomes greatly affects liposomal membrane fluidity. Changes in the FTIR spectrum intensities and DTA profiles were indicative of those in the gauche conformation of the hydrocarbon chain of the phospholipid, and of increased calcein release from liposomes which indicate the fluidity of the bilayer membrane of the liposomes. These results suggest that further studies on designing a theoretical model for enhancing liposome stability, either by using optimizing liposome formulation or other strategies to reduce membrane fludity, are imperative to support the development of strategies for the liposomal delivery of drugs targeting hepatic stage malaria.

## Methods

### Materials

Primaquine bisphosphate (PQ) was purchased from Sigma-Aldrich (Rehovot, Israel), while Chloroquine diphosphate (CQ) was obtained from Sigma-Aldrich (Gyeonggi-do, South Korea). Hydrogenated soybean phosphatidylcholine (HSPC) and 1,2-distearoyl-*sn*-glycero-3-phosphoethanolamine-N-(methoxy[polyethyleneglycol]-2000) (DSPE-mPEG_2000_), with an average molecular weight of 2800, were procured from Nof Corporation (Tokyo, Japan). Cholesterol was obtained from Wako Inc., Ltd. (Osaka, Japan). Calcein was acquired from Nacalai Tesque Inc. (Kyoto, Japan). KH_2_PO_4_, Na_2_HPO_4_, chloroform, and methanol were purchased from Merck Inc. (Darmstadt, Germany). Sephadex® G-50 was obtained from Sigma-Aldrich (Steinheim, Germany). Other reagents and materials used were of the finest grade available.

### Preparation of liposomes

The liposomes were prepared by means of thin-film hydration^26^ using the formula listed in Table 3. All lipid components, including HSPC, cholesterol, and DSPE-mPEG_2000_, were initially dissolved in chloroform before being homogenously mixed in a round-bottom flask. The organic solvent was subsequently evaporated using a rotary evaporator to form a thin lipid layer which was then hydrated with citrate buffer (pH 5.0) and extruded to produce liposomes of 100 nm in size. The extrusion process consisted of passing through three membranes with various pore sizes; the first with a pore size of 400 nm, the second with a pore size of 200 nm and the third with a pore size of 100 nm. Each step involved passing liposomes through a polycarbonate membrane in 30 repeated cycles by means of an extruder kit with a heating block (Avanti® Mini–Extruder, Avanti Polar Lipid Inc., Alabama, USA) at 55–60 °C. Furthermore, each drug was consequently loaded using a pH gradient method. The liposomal outer phase was replaced by passing the liposomes through a Sephadex® G-50 column saturated with phosphate-buffered saline (PBS, pH 7.4). The mixture was then mixed with PQ and CQ solution and incubated for 20 min at 60 °C. This was followed by separation of the PQ- and CQ-loaded liposomes from the free drugs using a Sephadex® G-50 column (Sigma-Aldrich).

**Table 3:** Formulation of blank and drug-loaded liposomes.

Component	Formulation			
	Lipo-Blank	Lipo-PQ	Lipo-CQ	Lipo-PQCQ
PQ	–	1.00	–	1.66 mg
CQ	–	–	3.33 mg	1.66 mg
HSPC	5.94 mg	5.94 mg	5.94 mg	5.94 mg
DSPE-mPEG_2000_	1.94 mg	1.94 mg	1.94 mg	1.94 mg
Cholesterol	2.13 mg	2.13 mg	2.13 mg	2.13 mg

*PQ* primaquine, *CQ* chloroquine, *HSPC* hydrogenated soybean phosphatidylcholine, *DSPE-mPEG2000* distearoyl-*sn*-glycero-3-phosphoethanolamine-N-(methoxy[polyethylene glycol]-2000).

The molar ratio of HSPC:DSPE-mPEG2000:cholesterol was 59:5:36. Primaquine and chloroquine were added by considering the weight ratios of the drugs to the lipid components of the liposomes, which were 1:10 for primaquine:total lipid in primaquine-loaded liposomes (Lipo-PQ), 1:3 for chloroquine:total lipid in chloroquine-loaded liposomes (Lipo-CQ), and 1:1:6 for primaquine:chloroquine:total lipid in liposomes loaded with primaquine and chloroquine (Lipo-PQCQ).

### Determination of particle size and ζ-potential of liposomes

The preparation was evaluated for particle size and polydispersity index (PDI) via dynamic light scattering, and ζ-potential was determined via electrophoresis light scattering using a Delsa™ Nano C Particle Analyzer at room temperature (25 °C). Approximately 100 µl of liposomes were diluted with 3 ml of distilled water and then placed into a cuvette to determine the particle size, PDI, and ζ-potential.

### Evaluation of encapsulation efficiency and drug loading capacity

After PQ, CQ, and their combination (PQCQ) had been loaded into liposomes, the mixtures were eluted through a Sephadex® G-50 column with PBS 7.4 to separate free drugs from their encapsulated counterparts. The samples were then lysed with methanol (50%, v/v), with PQ and CQ subsequently being determined by means of UV spectrophotometric method as previously reported^26^.

The encapsulation efficiency (EE) and loading capacity (LC) were calculated using Eqs. (1) and (2) respectively^26,38^:

1 $$EE\left( \% \right) = \frac{Amount\;of\;drug\;encapsulated}{{Amount\;of\;drug\;encapsulated + Amount\;of\;free\;drug }} \times 100,$$

2 $$LC\left( \% \right) = \frac{Amount\;of\;drug\;encapsulated}{{Total\;amount\;of\;drug + Total\;amount\;of\;liposomal\;components}} \times 100$$

### Spectroscopy and crystallography of the liposomes

#### Fourier-transform infrared (FTIR) spectroscopy of liposomes

The FTIR profiles of liposomes were analyzed using an FTIR spectrophotometer (Shimadzu, Kyoto, Japan). The freeze-dried liposomes were finely crushed and mixed with potassium bromide at a weight ratio of 1:100. The mixture was then pressed in a mechanical mold to form thin and translucent pellets, which were subsequently examined at wavenumbers of 4000–450 cm^−1^. The results of the infrared spectra obtained for the samples were compared with the literature values.

#### Powder X-ray diffraction (P-XRD) analysis of liposomes

P-XRD analysis was performed using a PRD instrument (Phillips X'Pert PRO PANalytical, Netherlands). Freeze-dried liposomes were placed in a container and flattened. This process was performed under the following conditions: Cu metal anode, Kα filter, voltage of 40 kV, 30 mA, and 2ø of 5°–90°.

#### Differential thermal analysis (DTA) of liposomes

A DTA instrument (Mettler Toledo FP 85, Switzerland) was used to perform a DTA. The dried liposomes were placed in aluminum crucibles and subsequently heated from 30 to 300 °C at a rate of 5 °C/min.

#### Nuclear magnetic resonance (NMR) analysis of liposomes

The ^1^H NMR spectra of blank liposomes (Lipo-Blank) and liposomes loaded with PQ (Lipo-PQ), CQ (Lipo-CQ), and both drugs (Lipo-PQCQ) were analyzed using a JEOL 400 ECA spectrophotometer (JEOL, Tokyo, Japan) at 400 MHz^53^. Approximately 5 mg of freeze-dried samples were dissolved in CDCL_3_ to produce a concentration of 10 mg/ml with the data integration subsequently analyzed by computer using JEOL Delta v5.04.

### Calcein release test as an indicator of membrane leakage

#### Preparation of calcein-loaded liposomes

The liposomes were composed of HSPC, cholesterol, and DSPE-mPEG_2000_ at a molar ratio of 55:40:5 using the thin-layer method. The thin lipid layer formed was then hydrated with a citrate buffer pH 5.0 containing 17 mM calcein, followed by the extrusion process using a 100-nm polycarbonate membrane. At the next stage, the calcein-loaded liposomes were separated from free calcein by passing the liposomes through a Sephadex® G-50 column saturated with PBS. The eluted liposomes were mixed with PQ and CQ solution, followed by incubation at 60 °C for 20 min. Finally, to obtain liposomes loaded with calcein and PQ and/or CQ, the liposomes were passed through a Sephadex® G-50 column saturated with PBS.

#### Calcein release study

Calcein release was studied using the dialysis method^24^. Liposomes containing equivalent amounts of 2 mM calcein were inserted into the Spectra Por® 7 dialysis membrane with a MWCO of 3500 Da. PBS pH 7.4 was used as the release medium with an agitation speed of 400 rpm at 37 °C., Sampling was subsequently conducted after 0.5, 1, 2, 4, 8, 12, 24, and 48 h, with each sample replaced with the same volume of PBS pH 7.4, heated at 37 °C. The cumulative amount of calcein released from liposomes was determined using a GloMax®-Multi + Detection System (Promega) in the fluorescence mode at λex = 490 nm and λem = 530 nm^54^.

Because of medium dilution during the release test procedure, the quantified amount of calcein was corrected using the dilution factor contained in Eq. (3), as follows^55^:

3 $$Cn = C^{\prime}n + \frac{a}{b}\mathop \sum \limits_{i = 1}^{n - 1} Cs$$

Description: Cn: measured percent drug release at time point n after correction; C′n: measured percent drug release at time point n before correction; Cs: measured percent drug release at time point n − 1; a: volume of the obtained sample (ml); b: volume of released medium (ml).

### Statistical analysis

All data were obtained from three replicates and presented as the mean ± SD. In addition, differences were further analyzed using one-way analysis of variance followed by the least significant difference test. Significance was indicated by *p* < 0.05.

### Ethical conduct of research statement

This article does not contain any studies with human and animal subjects performed by any of the authors.

## Supplementary Information

Supplementary Information.
