# Supplementary material for: Interactions of primaquine and chloroquine with PEGylated﻿ phosphatidylcholine liposomes
Source: Sci Rep. 2021 Jun 14;11:12420. doi: 10.1038/s41598-021-91866-0 (PMC8203617; doi:10.1038/s41598-021-91866-0)
Supplement: Supplementary file 1 — Supplementary Information. [file 41598_2021_91866_MOESM1_ESM.pdf]

SUPPLEMENTARY INFORMATION FILE

Interactions of primaquine and chloroquine with PEGYlated phosphatidylcholine liposomes

Andang Miatmoko[1,*], Ira Nurjannah[1], Nuril Fadilatul Nehru[1], Noorma Rosita[1], Esti Hendradi[1], Retno Sari[1], Juni Ekowati[1]

[1] Department of Pharmaceutical Sciences, Faculty of Pharmacy, Universitas Airlangga, Nanizar Zaman Joenoes Building, Campus C Unair, Mulyorejo, 60115, Indonesia

Running Title: Interactions of primaquine and chloroquine with phosphatidylcholine liposomes

[*] To whom correspondence should be addressed:
E-mail address: andang-m@ff.unair.ac.id
Tel/fax: +62-31-5933-150/+62-31-5935-249

SUPPLEMENTARY INFORMATION

Analysis of liposomes morphology

A drop of liposome was applied to a carbon-coated copper grid and left for a minute to allow particles to adhere to the carbon substrate. The excess dispersion of liposomes was then removed with a piece of filter paper. A drop of a 1% uranyl acetate solution was applied to the sample for one minute before being allowed to air-dry. Finally, the samples were observed with a transmission electron microscope (JEOL JEM-1400).

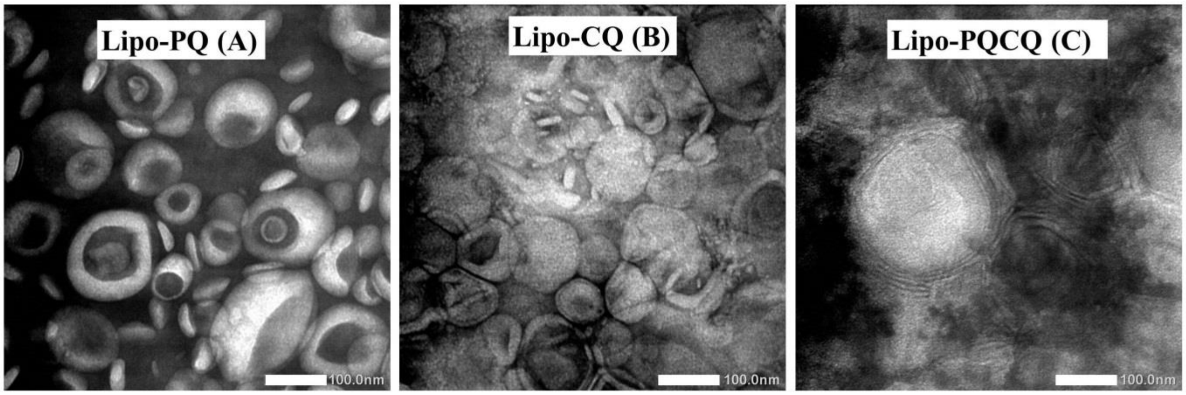

Supplementary Figure 1 Transmission electron microscopy (TEM) images of primaquine-loaded liposomes (Lipo-PQ), chloroquine-loaded liposomes (Lipo-CQ), and liposomes loaded with both primaquine and chloroquine (Lipo-PQCQ) stained with uranyl acetate. The scale bar is 100 nm.
